# Fibre Morphological Characteristics of Kraft Pulps of *Acacia melanoxylon* Estimated by NIR-PLS-R Models

**DOI:** 10.3390/ma9010008

**Published:** 2015-12-25

**Authors:** Helena Pereira, António José Alves Santos, Ofélia Anjos

**Affiliations:** 1 Centro de Estudos Florestais, Instituto Superior de Agronomia, Universidade de Lisboa, Tapada da Ajuda, 1349-017 Lisboa, Portugal; hpereira@isa.ulisboa.pt (H.P.); antonioalvesantos@gmail.com (A.J.A.S.); 2 Instituto Politécnico de Castelo Branco, Escola Superior Agrária, Apartado 119, 6001-909 Castelo Branco, Portugal

**Keywords:** *Acacia melanoxylon*, Kraft pulps, fibre length and width, NIR

## Abstract

In this paper, the morphological properties of fiber length (weighted in length) and of fiber width of unbleached Kraft pulp of *Acacia melanoxylon* were determined using TECHPAP Morfi^®^ equipment (Techpap SAS, Grenoble, France), and were used in the calibration development of Near Infrared (NIR) partial least squares regression (PLS-R) models based on the spectral data obtained for the wood. It is the first time that fiber length and width of pulp were predicted with NIR spectral data of the initial woodmeal, with high accuracy and precision, and with ratios of performance to deviation (RPD) fulfilling the requirements for screening in breeding programs. The selected models for fiber length and fiber width used the second derivative and first derivative + multiplicative scatter correction (2ndDer and 1stDer + MSC) pre-processed spectra, respectively, in the wavenumber ranges from 7506 to 5440 cm^−1^. The statistical parameters of cross-validation (RMSECV (root mean square error of cross-validation) of 0.009 mm and 0.39 μm) and validation (RMSEP (root mean square error of prediction) of 0.007 mm and 0.36 μm) with RPD_TS_ (ratios of performance to deviation of test set) values of 3.9 and 3.3, respectively, confirmed that the models are robust and well qualified for prediction. This modeling approach shows a high potential to be used for tree breeding and improvement programs, providing a rapid screening for desired fiber morphological properties of pulp prediction.

## 1. Introduction

Pulp is a major global commodity forest-based product that consists of the dissociated cells of the raw material, usually named pulp fibers. The fiber morphological properties are important quality parameters for pulp and paper properties and therefore they are measured both for research purposes and industrial quality control.

In fact, the fiber characteristics greatly influence the quality and properties of the final product, e.g., they are frequently correlated with the physical and mechanical properties of paper and paperboard [1,2,3,4,5,6]. For instance, strong correlation between fiber length and tear index was reported for both hardwoods and softwoods [7,8,9,10,11]. Tensile and tear strength of paper increase with fiber length, especially in weakly bonded sheets [12,13]. Fiber length influences the papersheet formation and its uniformity [13,14].

Fiber biometry has also been found correlated with other pulp variables. For instance, the pulp yield correlated positively with fiber length and negatively with fiber width [15] and Wimmer *et al.* [16] reported that fiber length of *E. globulus* had a strong effect on pulp yield and freeness, as well as active alkali consumption in addition to tear index and bending stiffness.

Fiber morphological variables, namely length and width, are among the main fundamental characteristics of the pulp fibers. Therefore, methods for measuring fiber morphology are essential for determining pulp quality.

The traditional method involves classifying the pulp into screened fractions [17], measuring the weight and length of fibers in each fraction to calculate the weighted average length by weight [18]. Automated optical analyzers are now used [19] such as Kajaani FS300 (Metso Automation Inc., Helsinki, Finland), OpTest HiRes FQA (OpTest Equipment Inc., Hawkesbury, ON, Canada), Fiber Lab (Metso Automation Inc., Helsinki, Finland), TECHPAP Morfi lab (Techpap SAS, Grenoble, France), L & W STFI Fibermaster (Lorentzen & Wettre, Stockholm, Sweden). In spite of their relatively easy operation, these methods require the measurement of a large number of fibers from a pulp suspension, and are therefore not time- and cost-effective when a large number of wood samples have to be tested.

This is the case, for instance, in screening programs of pulping raw materials, namely in pulpwood improvement programs, for which fiber morphology is one of the quality traits. Given the high number of samples that have to be tested in such programs, it would be important to be able to predict fiber length and width in a faster way. The use of non-destructive measurements is tempting, especially the spectroscopic methodologies using the near infrared wavelength range (NIRS). The application of NIRS for predicting several properties of wood and pulps using statistical modeling tools, namely PLS models (Partial least squares), has already proved successful [20].

Comparatively few attempts were made to develop NIRS-based models for the morphological variables of wood. Schimleck *et al.* [21] examined the use of NIR spectroscopy for predicting tracheid length of loblolly pine wood and considered that the accuracy was sufficient for ranking purposes. NIRS-based predictions of tracheid coarseness and wall thickness were excellent [22]. Jones *et al.* [23] and Schimleck *et al.* [21] also reported the effect of green wood condition and a wide range of sites on the estimate of tracheid morphological characteristics of loblolly pine wood by NIR spectroscopy.

Via *et al.* [24] studied the variation of tracheid length in longleaf pine with tree age and height using NIR-based prediction. Wang *et al.* [25] established an NIR-based PLS-R model to predict fiber length of slash pine and poplar. Viana *et al.* [26] also predicted the morphological characteristics and basic density of eucalypt clones wood using NIR, but the best calibration correlations were obtained for basic density. Inagaki *et al.* [27] predicted fiber length for *Eucalyptus camaldulensis* with an RPD (ratios of performance to deviation) of 3.8 from solid wood samples using NIR-based PLS-R models. Sun *et al.* [28] estimated the MFA (microfibril angle) and fiber length of bamboo by NIRS, and the PLS models based on noise combined with orthogonal signal selection spectra gave the strongest correlations.

The prediction of pulp fiber morphology using NIRS of the wood has not been attempted. This is made in the present work, which focuses on the development of a NIR-PLS-R model for the prediction of fiber length and width in the unbleached Kraft pulps using *Acacia melanoxylon* wood meal as a case study. *A. melanoxylon* is a valuable timber species, but is also of interest for pulping as evaluated regarding pulp yield and properties [15,29,30,31]. Pulps produced from Acacia species are competitive in the world market of hardwood pulps now dominated by *Eucalyptus* [32].

In this work the pulps were produced under identical pulping conditions, as would be the case in a screening program or in pulp mill operation, and the measurements of fiber length weighted in length and fiber width used for the modeling were determined with TECHPAP Morfi^®^ equipment (Techpap SAS, Grenoble, France). We aimed to obtain models performing better than those found in the literature, which are not precise enough for screening purposes, according to the AACC (American Association of Cereal Chemists) Method 39-00 [33], due to failing the ratios of performance to deviation (RPD) criteria.

## 2. Results

The fiber length and width of the unbleached pulps of *A. melanoxylon* obtained in this study ranged from 0.66 to 0.79 mm and 16.4 to 22.3 μm, respectively, with an average of 0.73 ± 0.03 mm and 18.8 ± 1.4 μm (Table 1). This variability represents a good data scattering and the two sets showed similar statistics.

**Table 1:** Number of samples and range of fibre length (weighted in length) and width in the calibration set and test set, with average, standard deviation (SD) and coefficient of variation (CV).

Parameter	Fiber Length (mm)		Fiber Width (μm)	
	Calibration Set	Test Set	Calibration Set	Test Set
Number of samples	45	15	45	15
Maximum	0.79	0.79	22.3	21.6
Minimum	0.66	0.69	16.4	16.7
Average	0.73	0.73	18.7	18.9
SD	0.03	0.03	1.4	1.2
CV	4.0	3.7	6.4	5.6

The near-infrared data from the 45 samples of the calibration set were regressed against their experimentally determined fibre length weighted in length and width, and the results obtained with the various pre-processing of the raw spectral data are summarized in Table 2. All pre-processing was reported in Table 2 in order to compare the choice model with the other tested models. In this study, the spectral information in the wavenumber range from 7506 to 5440 cm^−1^ was used for calibration, as found by automated optimization.

It is interesting to notice that the wavenumber range varies greatly with the parameter being studied. For instance, NIR-PLS-R models published for different parameters of *A. melanoxylon* used different wavenumber ranges, as summarized in Table 3.

The calibration for fiber length obtained a rank ranging from eight (2ndDer—seconded derivative, 1stDer + MSC—first derivative + multiplicative scatter correction, 1stDer + VecNor—first derivative + vector normalization) to 10 (ConOff—constant offset elimination, no spectral pre-processing), while the coefficients of determination (*r*^2^) ranged from 80.2% to 93.4%, and RMSECV (root mean square error of cross-validation) from 0.008 to 0.014 mm. For fiber width, the obtained calibrations ranks ranged from six (1stDer + MSC) to 10 (no spectral pre-processing), with the coefficients of determination (*r*^2^) ranging from 88.5% to 93.0%, and RMSECV from 0.37 to 0.45 μm. When using the test set validation for fiber length and fiber width, the coefficients of determination ranged from 62.4% to 93.5% and 72.3% to 93.7%, respectively.

The best model was selected by using the 2ndDer (second derivative) for fiber length and 1stDer + MSC for fiber width of the spectral data. The 2ndDer pre-processing for the classification of length was selected instead of 1stDer + SLS (first derivative + straight line subtraction) because the first one better corrects the effects of wood grain than the 1stDer + SLS. The capacity of the prediction by NIR of morphological properties of fibers is a consequence of the effect of the materials density which results from the arrangement of the particles in the structure, in this specific case the length and width of the fibers. Thus, when used in the diffuse reflectance mode in spectrum acquisition, those are quantified amounts of light unabsorbed by materials.

**Figure 1:**
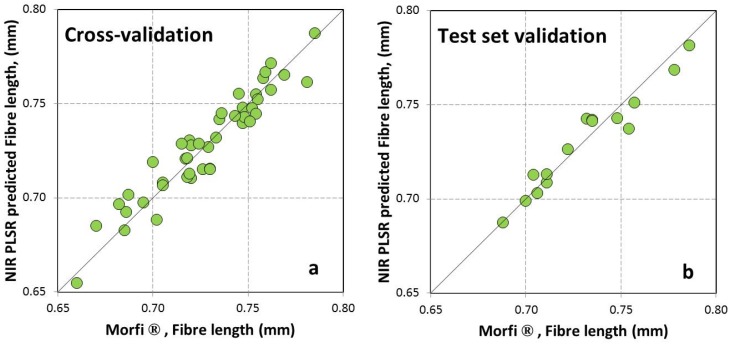

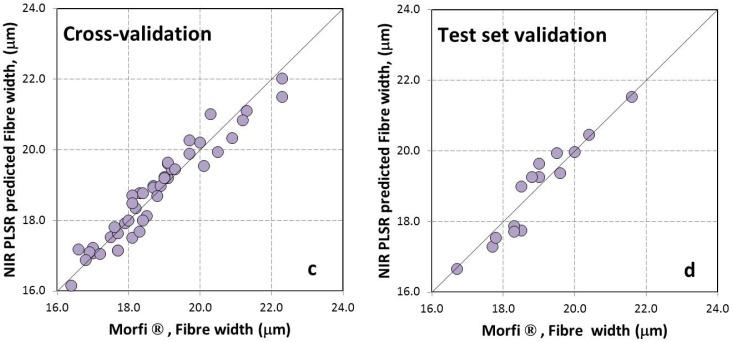
NIR-PLS-R predicted *versus* laboratory determined fiber length and fiber width for *Acacia melanoxylon* pulps for the cross-validation (**a**,**c**) and test set validation (**b**,**d**).

**Figure 2:**
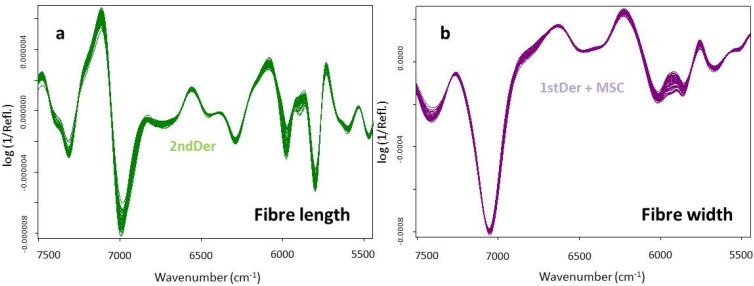
NIR diffuse reflectance for all spectra (45 samples—cross-validation) of the fiber length and fiber width for *Acacia melanoxylon* pulps (**a**,**b**, respectively).

The corresponding plot of NIR-PLS-R predicted *versus* the laboratorial determined fiber length and fiber width is shown in Figure 1. Both the cross-validation and the validation showed high correlation between predicted and determined values, with a RMSEP of 0.007 mm and 0.36 μm, a rank of eight and six, and no outliers respectively for fiber length and fiber width. Figure 2 shows NIR diffuse reflectance spectra of the fiber length and fiber width for the 2ndDer and 1stDer + SLS preprocessing used, respectively.

**Table 2:** Results of the cross-validation of the calibration set (45 samples) and of the test set validation (15 samples) including percentage of outsiders (OS) and outliers (OL) of the test set obtained during prediction of the samples with unknown fiber length (weighted in length) and width, using various pre-processing methods of the raw spectral data. In bold: cross-validation for the best model with test set validation.

Parameter	Pre-Processing	Number of Samples	Cross-Validation (CV)				Test Set Validation (TS)					
			Rank	*r*^2^ (%)	RMSECV	RPD	Rank	*r*^2^ (%)	RMSEP	RPD	OS (%)	OL (%)
Fiber Length (mm)	1stDer	45	9	92.4	0.008	3.6	8	87.8	0.010	2.9	0.0	0.0
	**2ndDer**	**45**	**8**	**91.6**	**0.009**	**3.5**	**8**	**93.5**	**0.007**	**3.9**	**0.0**	**0.0**
	ConOff	44	10	90.6	0.009	3.3	9	76.9	0.014	2.2	0.0	0.0
	MinMax	45	9	80.2	0.014	2.3	8	76.6	0.014	2.1	0.0	0.0
	MSC	45	9	81.7	0.013	2.3	7	70.9	0.015	1.9	0.0	0.0
	None	42	10	88.5	0.010	3.0	9	62.4	0.017	1.8	0.0	0.0
	SLS	45	9	91.7	0.009	3.5	7	80.1	0.013	2.3	0.0	0.0
	VecNor	45	9	81.6	0.013	2.3	7	70.7	0.015	1.9	0.0	0.0
	1stDer + MSC	44	8	86.5	0.011	2.7	6	76.4	0.014	2.1	0.0	0.0
	1stDer + SLS	45	9	93.4	0.008	3.9	7	88.9	0.009	3.0	0.0	0.0
	1stDer + VecNor	44	8	86.4	0.011	2.7	6	76.4	0.014	2.1	0.0	0.0
Fiber Width (μm)	1stDer	45	7	91.8	0.41	3.5	6	85.0	0.45	2.6	0.0	0.0
	2ndDer	44	7	88.5	0.45	3.0	9	93.7	0.29	4.0	0.0	0.0
	ConOff	45	9	92.6	0.38	3.7	8	71.1	0.62	1.9	0.0	0.0
	MinMax	45	8	92.5	0.39	3.7	7	75.1	0.58	2.0	0.0	0.0
	MSC	45	8	93.0	0.37	3.8	6	75.0	0.58	2.0	0.0	0.0
	None	45	10	92.6	0.38	3.7	5	75.7	0.51	2.1	0.0	0.1
	SLS	45	8	92.5	0.37	3.7	7	72.3	0.61	1.9	0.0	0.0
	VecNor	45	8	93.0	0.37	3.8	6	74.7	0.58	2.0	0.0	0.0
	**1stDer + MSC**	**45**	**6**	**92.5**	**0.39**	**3.7**	**6**	**90.4**	**0.36**	**3.3**	**0.0**	**0.0**
	1stDer + SLS	44	7	90.2	0.41	3.2	7	88.2	0.40	2.9	0.0	0.0
	1stDer + VecNor	45	6	92.5	0.39	3.7	6	87.6	0.41	2.9	0.0	0.0

Rank—number of PLS components; *r*^2^—coefficient of determination; RMSECV—root mean square error of cross-validation; RPD—ratios of performance to deviation; RMSEP—root mean square error of prediction; 1stDer—first derivative; 2ndDer—seconded derivative; ConOff—constant offset elimination; MinMax—minimum-maximum normalization; MSC—multiplicative scatter correction; None—no spectral data processing; SLS—straight line elimination; VecNor—vector normalization; 1stDer + MSC—first derivative + multiplicative scatter correction; 1stDer + SLS—first derivative + straight line subtraction; 1stDer + VecNor—first derivative + vector normalization.

**Table 3:** Wavenumber ranges and pre-processing used in the NIR-PLS-R (infrared (**NIR**) partial least squares regression (**PLSR**) models published in bibliography for different properties of *Acacia melanoxylon*.

Property	References	Material	Wavenumber Ranges (cm^−1^)	Pre-Processing
Wood basic density	[34]	Solid wood	8250–7494 + 5450–5304	1stDer + SLS
Pulp yield	[35]	Woodmeal	9087–5440 + 4605–4243	2ndDer
Kappa number	[36]	Kraft pulp	6110–5440	1stDer
ISO brightness of unbleached pulp	[37]		9404–7498 + 4605–4243	1stDer

## 3. Discussion

When considering all the samples of this study (60 samples), a good calibration for the fiber length and fiber width of the unbleached Kraft pulp was obtained with the spectral data of the woodmeal with *r*^2^ = 96.5% and 95.0%, Rank = 8 and 6, root-mean-square error of estimation (RMSEE) = 0.006 mm and 0.32 μm and RPD = 5.3 and 4.5, respectively (Table 4). These results compare very favorably with the few data available for the estimation of morphological properties of wood using NIR spectroscopy, as shown in Table 5.

**Table 4:** Results of the calibration and cross-validation for all samples (60 samples) for fiber length (weighted in length) and width of *Acacia melanoxylon* pulps, using the best pre-processing methods of the raw spectral data.

Parameter	Pre-Processing	Calibration (C)				Cross-Validation (CV)			
		Rank	*r*^2^ (%)	RMSEE	RPD	Rank	*r*^2^ (%)	RMSECV	RPD
Fiber length (mm)	2ndDer	8	96.5	0.006	5.3	8	94.0	0.007	4.1
Fiber width (μm)	1stDer + MSC	6	95.0	0.32	4.5	6	92.8	0.37	3.7

Rank—number of PLS components; *r*^2^—coefficient of determination; RMSECV—root mean square error of cross validation; RPD—ratios of performance to deviation; RMSEE—root mean square error of estimation; 2ndDer—seconded derivative; 1stDer + MSC—first derivative + multiplicative scatter correction.

For instance, the models for tracheid radial and tangential diameter of softwoods [21,22,23] were weaker with *r*^2^ from 45.0% to 80.0%, standard error of calibration (SEC) from 0.60 to 1.60 μm, and the validation of the models showed higher standard error of prediction (SEP) from 1.04 to 2.70 μm.

Inagaki *et al.* [27] proposed (*Eucalyptus camaldulensis*) one model for the prediction of fiber length in wood for hardwoods, with a root mean square error of cross-validation (RMSECV) of 0.018 mm and 0.012 mm to a root mean square error of prediction (RMSEP), respectively, with *r*^2^ 87.0% and 93.0%.

The ratios of performance to deviation (RPD) may be used to evaluate if the prediction models fulfill the requirements of the AACC Method 39-00 for screening in breeding programs that require a RPD ≥ 2.5 [33]. The RPD was introduced by Williams and Norris [38] as the ratio between the standard deviation of the reference data of the validation set and the standard error of prediction of a cross-validation or of the test set validation. In the present case, the RPD for the validation of the NIR-PLS-R model was 3.9 and 3.3, respectively, for fiber length weighted in length and fiber width (Table 2), thereby allowing the conclusion that it is applicable for screening in breeding programs.

It should be stressed that the fiber biometric characteristics of the unbleached pulp can be accurately predicted by the spectral data of the unprocessed (*i.e*., unpulped) wood material. The practical interest for improvement and breeding programs is obvious, and the application of NIRS-PLS-R models will allow a very high reduction in cost and time for the experimental evaluation.

**Table 5:** Model characteristics for some cellular morphological properties of wood using NIR spectroscopy published in the bibliography.

Species	Reference	Parameter	Pre-Processing	Rank	*r*^2^	RMSECV	RMSEP
*Eucalyptus camaldulensis*	[27]	Fiber length (mm)	MSC	6	87.0/93.0	0.018 mm	0.012 mm
*Pinus taeda*	[23]	Tracheid radial diameter (μm)	1stDer (a)	10	46.0/42.0	1.58 * μm	1.77 ** μm
			1stDer (b)	10	45.0/46.0	1.59 * μm	1.60 ** μm
		Tracheid tangential diameter (μm)	1stDer (a)	6	60.0/63.0	1.00 * μm	1.09 ** μm
			1stDer (b)	6	60.0/62.0	1.02 * μm	1.04 ** μm
	[21]	Tracheid radial diameter (μm)	2ndDer (c)	6	70.0/36.0	1.10 * μm	1.50 ** μm
		Tracheid tangential diameter (μm)		6	80.0/61.0	0.60 * μm	1.30 ** μm
		Tracheid radial diameter (μm)	2ndDer (d)	5	59.0/16.0	1.30 * μm	1.70 ** μm
		Tracheid tangential diameter (μm)		4	73.0/16.0	0.70 * μm	1.70 ** μm
*Pinus radiata*	[22]	Tracheid radial diameter (μm)	2ndDer	6	65.0/28.0–30.0	1.60 * μm	1.90–2.70 ** μm
		Tracheid tangential diameter (μm)		4	69.0/69.0–79.0	1.00 * μm	1.20–1.70 ** μm

(a) original calibration; (b) original calibration with new data; (c) spectra on radial-longitudinal face of wood samples; (d) spectra on the transverse face of dry wood samples; * value corresponding to standard error of calibration (SEC); ** value corresponding to standard error of prediction (SEP).

## 4. Materials and Methods

### 4.1. Wood Samples

A total of 60 wood discs from *Acacia melanoxylon* R. Br., belonging to 20 trees from four sites in Portugal and collected at different stem height levels, were used in this study. Detailed information on samples, sites and stands is available elsewhere [39].

Wood meal samples were prepared by milling using a knife mill (Retsch) with a 1 mm output screen and the fraction coarser than 0.25 mm was retained for spectral acquisition.

### 4.2. Kraft Pulps

Samples of 25 g oven-dry woodmeal were Kraft pulped using a multi-batch digester system under the following reaction conditions that were set to obtain a target kappa number of 15: active alkali charge 21.3% (as NaOH); sulfidity 30%; liquor/wood ratio 4/1; time to temperature of 160 °C, 90 min; time at temperature of 160 °C, 90 min. The pulped samples were disintegrated, washed, and screened. Under these conditions the pulp yields ranged from 47.0% to 58.2% [15,35], as related to the variation of heartwood proportion in different wood discs [39].

### 4.3. Morphological Properties of Pulp Fibers

The morphological properties (fiber length—weighted in length—and width) of the unbleached pulps of *A. melanoxylon* were determined automatically by image analysis of a diluted suspension (20 mg L^−1^) in the flow chamber of TECHPAP Morfi^®^ Equipment, by measuring at least 5000 fibers. The morphological properties of the pulp fibers used in this study are the same samples determined by Santos *et al.* [15] according to TAPPI 271 pm-98 [19].

The fiber length weighted in length (*L_w_*—(Equation (1))) and fiber width (*l_N_*—(Equation (2))) are calculated by TECHPAP Morfi^®^ as:

(1) $$L_{w}=\frac{\sum_{i=0}^{N}\;\left(Li\times Li\right)}{\sum_{i=0}^{N}Li}$$

(2) $$l_{N}=\frac{\sum_{i=0}^{N}li}{N}$$

where *N* is the number of fiber, *i* is fiber *i*, *Li* is the average length weighted in length, and *li* width of the fiber *i*.

The experimental error using the TECHPAP Morfi^®^ in the measurements of the fiber length—weighted in length—and width was 0.5% and 1%, respectively.

### 4.4. Spectra Collection and Data Processing

The woodmeal samples were conditioned in a climatic chamber at 60 °C for a period of 48 h before spectral acquisition. NIR spectra were collected in the wavenumber range from 12,000 to 3800 cm^−1^ with a near infrared spectrometer (BRUKER, model Vector 22/N, Karlsruhe, Germany) in diffuse reflectance mode, using a spinning cup module. Each spectrum was obtained with 100 scans at a spectral resolution of 16 cm^−1^. After collecting the spectra, the woodmeal samples were used for production of the Kraft pulps.

The samples were randomly divided into a calibration set containing 45 samples and a validation set (test set) containing 15 samples. The processing was done in two steps. First, the infrared data from the calibration samples were regressed against the measured fiber length and width, and by means of full cross-validation with one sample omitted a significant number of PLS components (rank) was obtained using OPUS/Quant 2 software (version 7.5.18 BRUKER, Bruker Corporation, Karlsruhe, Germany). Besides the raw spectra, also pre-processed spectra with 10 methods were used for PLS analysis [36,37,40]. In a second step, the validation of the PLS-R models was performed using the independent test set. The number of PLS factors was found by automated optimization.

The quality of the calibration models was assessed by means of cross-validation and by using the test set validation results by determining their coefficient of determination (*r*^2^), root mean square error of cross-validation (RMSECV), root mean square error of prediction (RMSEP) and the residual prediction deviation or ratio of performance to deviation (RPD).

The selection of the final model was based on its predictive ability assessed by the least possible number of samples classified as outsiders and/or outliers.

## 5. Conclusions

NIRS-PLS-R models could be developed to predict the biometric characteristics of fiber length and width of unbleached Kraft pulps using the spectral data of the initial wood meal. The statistical parameters of cross-validation (RMSECV of 0.009 mm and 0.39 μm) and validation (RMSEP of 0.007 mm and 0.36 μm) with RPD_TS_ values of 3.9 and 3.3, respectively, confirm that the models are robust, stable, and well qualified for prediction.

This modeling approach using NIR spectral data of wood to predict pulp fiber dimensions was presented here for the first time. It has a high potential to be used for tree breeding and improvement programs by providing a rapid screening for desired fiber morphological properties of pulp.
